# *In vitro* biocompatibility analysis of functionalized poly(vinyl chloride)/layered double hydroxide nanocomposites

**DOI:** 10.1039/c8ra06175k

**Published:** 2018-12-05

**Authors:** Monika Singh, Rajesh Kumar Singh, Santosh Kumar Singh, Sanjeev Kumar Mahto, Nira Misra

**Affiliations:** School of Biomedical Engineering, Indian Institute of Technology, (Banaras Hindu University) Varanasi 221005 India nira1953@yahoo.com; Centre of Experimental Medicine and Surgery, Institute of Medical Science, Banaras Hindu University Varanasi 221005 India

## Abstract

The aim of this study was to examine the cytotoxicity and biocompatibility of functionalized poly(vinyl chloride) (PVC)/layered double hydroxide (LDH) nanocomposites. The biocompatibility of the LDH-based nanocomposites of thiosulphate PVC (TS-PVC), thiourea PVC (TU-PVC) and sulphite PVC (S-PVC) was assessed *via* haemolysis and thrombogenicity tests followed by the analysis of cellular adhesion and proliferation. The MTT assay was performed on cells in direct contact with the polymeric nanocomposites to evaluate the side effects of the biomaterials. The cellular morphology of mouse mesenchymal stem cells was also analyzed after incubation with direct contact with the functionalized polymer nanocomposites for different time periods. Although the results of the haemolysis test displayed a positive influence of LDH on the functionalized PVC compared to the neat PVC, the thrombogenic property was observed to be notably decreased, which indicated improved blood compatibility. The resulting LDH samples were also studied for their performance *via* fluorescence imaging of cells after incubation with the materials. The LDH-based polymers exhibited an excellent level of cytocompatibility, which validates their use as biomaterials. PVC-TU/LDH-2 and PVC-S-2 were found to be notably less cytotoxic for the tested cell type. Also, the cells were found to adhere better to the entire PVC-S/LDH nanocomposite surface. The cytotoxicity test also revealed that the PVC-TU/LDH and PVC-S/LDH nanocomposites exhibited similar responses. The fluorescence-based image analysis showed that cells were spread much more on the polymer surface containing a higher LDH weight percentage. Overall, this study provides a benchmark for the biocompatibility properties of PVC/LDH nanocomposites, which may be useful for numerous applications in the biomedical and related areas.

## Abstract 2

The aim of this study was to examine the cytotoxicity and biocompatibility of functionalized poly(vinyl chloride) (PVC)/layered double hydroxide (LDH) nanocomposites.

## Graphical abstract

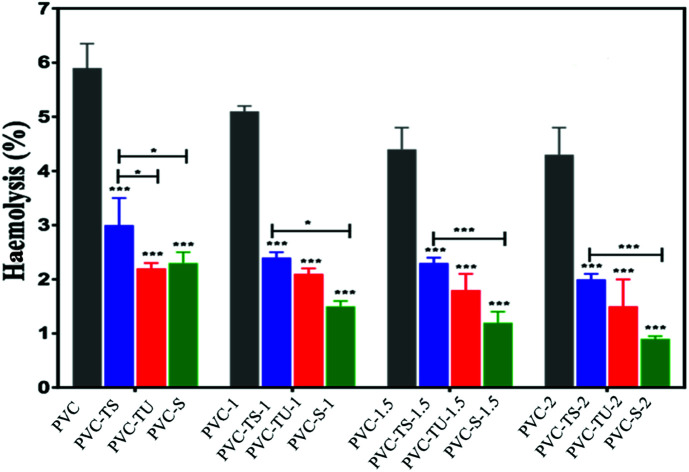

## Introduction

Synthetic polymers as biomaterials have been extensively used in the biomedical field due to their good elasticity and favorable mechanical and chemical properties.^1,2^ Additionally, these polymers are used as bioinert materials to restore tissue functionality. After the implantation of any material in living tissue, physiological incompatibility may occur as a result of the interaction between the synthetic material and the adjacent cellular environment. Thus, several approaches have been investigated to modify these materials for improving their biocompatibility.^3,4^ It has been observed that the behavior of the adhesion and proliferation of different types of cells on polymeric materials depends on their surface characteristics such as wettability, chemistry, charge, roughness and rigidity. Accordingly, different methods are applied to increase the biocompatibility of polymeric materials, such as the addition of charged moieties or functional groups on their surfaces^5–7^

Layered double hydroxide (LDH) nanoclay is a class of two dimensional (2D) organized material that has been explored for different biomedical applications because it possesses controlled anion exchange properties,^8^ which make it a perfect choice for the present study. LDHs have the general formula M^II^_1−*x*_M^III^_*x*_(OH)_2_(A_*x*/*n*_^*n*−^·*m*H_2_O), where M^II^ is a divalent cation (Mg^2+^, M^2+^, Fe^2+^, Co^2+^, Ni^2+^, Cu^2^, Zn^2+^ and Ca^2+^), M^III^ is a trivalent ion (Al^3+^, Cr^3+^, Mn^3+^, Fe^3+^, Co^3+^, Ni^3+^ and La^3+^) and A^*n*−^ is a gallery anion such as Cl^−^, CO_3_^2−^, and NO_3_^−^.^9^ According to Liang *et al.*,^10^ LDH is considered to be the most biocompatible nanostructure among all the inorganic materials, and thus, has received considerable attention recently. Although, LDH was discovered by Swedish scientists in 1842,^11^ their controlled synthesis and diverse applications were studied by several other authors^12–21^ in terms of blood and cellular biocompatibility. Choy *et al.*^22–27^ intercalated DNA nucleotides into LDH layers for gene therapy and bio-sensing applications. Subsequently, other authors focused on different intercalated molecules such as amino acids,^28,29^ peptides,^30–37^ sorbic acid,^38^ biocatalysts,^39,40^ porphyrins^41^ and non-ionic pentose^42^ for diverse purposes and applications. In addition, Hwang *et al.*^29^ prepared two types of inorganic molecules, hydrozincite and layered double hydroxide, for the intercalation of functionalized organic molecules, such as retinoic acid, ascorbic acid, indole acetic acid, citric acid, salicylic acid, and acidic dye *via* co-precipitation for pharmaceutical, cosmeceutical and nutraceutical functions. Pharmaceutical technology requires formulations to maintain pharmacologically active drug levels for long periods, avoiding repeated administration, and to localize drug release at the pharmaceutical target. The interlayer region of LDHs may be considered as the carrier, in which an anionic drug may be intercalated and released *via* the deintercalation process^43^ for drug delivery,^44^ enhanced implant corrosion resistance,^45–47^ and cancer treatment.^48^

Several reports are available in the literature on the preparation of different polymer/LDH nanocomposites.^49–53^ In recent years, nanocomposites based on PVC have attracted increasing attention. PVC/montmorillonite-^54^ and PVC/cellulose-based^2^ nanocomposites have been studied. The effect of finely powdered CaCO_3_ on the impact resistance of PVC has also been researched,^4^ but few publications considering the effect of nano CaCO_3_ on the mechanical properties of PVC and PVC blends have been reported. In addition, they have the ability to afford the exfoliation/intercalation phenomenon, which enhances the mechanical properties.^55–59^ Wang *et al.*^60^ modified clay with three different surfactants to form polymer nanocomposites with polyurethane, and Corrales *et al.*^61^ prepared poly(ε-caprolactone)/montmorillonite nanocomposites for improving the biological properties of polymers.

Currently, the biocompatibility of biomedical equipment is a serious concern, which affects many areas such as the pharmaceutical field and healthcare products.^62,63^ To overcome this problem a method was proposed to modify a basic polymer with functional groups and intercalate an inorganic molecule that can hold it, which improved its properties, making it better than the original. The literature shows that LDH have high potential as containers for functional agents due to their high biocompatibility, high chemical stability, and controlled release rate.^64^ However, to date, only a few studies have been reported on the application of LDH in the active functional polymer field.^65–67^ Therefore, it requires a fresh assessment and further research for further development.

The extensive review of previous works suggested that none of the earlier researchers used functionalized PVC for the preparation of PVC/LDH nanocomposites. Therefore, the aim of this study was to examine the effect of LDH and functionalized polyvinyl chloride (PVC)/LDH nanocomposites in terms of biocompatibility. Cytotoxicity tests were performed to examine the presence and release of leachable toxic materials and compounds. Also, the cells material interactions on the surface of the polymers were observed *via* fluorescence microscopy and analysis of the corresponding images.

## Experimental

### Synthesis of functionalized PVC/LDH nanocomposites films

Functionalization of PVC was performed using thiosulphate, thiourea and sulphite *via* the nucleophilic substitution method.^68^ To obtain the functionalized polymer, PVC was dissolved in THF and its prepared film was used as a control. For obtaining the modified PVC films, 10 g of PVC was dissolved in aqueous solutions of various solutes, including 3 M sodium thiosulphate, 7 M thiourea and 7 M sodium sulphite at room temperature. Then the solutions were heated at 60–65 °C and then tetrabutylammonium hydrogen sulphate (TBAHS) (0.15 M) was added in small amounts. The reaction mixture was kept at the same temperature for 5 h with continuous stirring. After 24 h, the solution was filtered and washed with double distilled water followed by methanol and dried under vacuum.

To obtain nanoclay, LDH was synthesized *via* the coprecipitation method^58^ with a 2 : 1 ratio of Mg : Al metal ions. Next, to acquire the polymer nanocomposites, the solution intercalation method was used.^56^ The functionalized PVC was dissolved in THF, and this solution was mixed with an LDH/THF sonicated solution and continuously stirred for 6 h at room temperature. THF was then allowed to evaporate and transparent films of all the functionalized PVC/LDH nanocomposites were finally obtained.

Henceforth, the notations of PVC, PVC-TS, PVC-TU, and PVC-S are used for the pure polymer and modified polymers. Furthermore, PVC-1, 1.5 and 2 represent the PVC/LDH composites, PVC-TS-1, 1.5 and 2 the PVC-TS/LDH nanocomposites, PVC-TU-1, 1.5 and 2 the PVC-TU/LDH nanocomposites and PVC-S-1, 1.5 and 2 the PVC-S/LDH nanocomposites.

### Blood biocompatibility

#### Haemolysis assay

The haemolytic activity of the various polymers was investigated according to the standard procedure described by Kapusetti *et al.*^69^ using acid citrate dextrose (ACD) human blood. Experiments were performed in accordance with the guidelines of the ICMR Government of India and approved by the ethics committee at IMS, Banaras Hindu University. Informed consent was obtained from non-alcoholic healthy donors. Blood was taken from healthy donors in accordance with the CAEC, University statement and consent was obtained from the participants. ACD blood (5 mL) was prepared by adding 4.5 mL of fresh human blood to 0.5 mL ACD. The ACD solution was prepared by mixing 0.544 g of anhydrous citric acid, 1.65 g of trisodium citrate dihydrate and 1.84 g of dextrose monohydrate in 75 mL of distilled water. The polymer films were cut into 0.5 × 0.5 cm square pieces and immersed in a phosphate buffered solution for 30 min at 37 °C in a desiccator. For the positive and negative controls, distilled water and a buffer solution were used, respectively. Thereafter, 0.2 mL ACD blood was added to each test tube, which was then kept for 1 h in an incubator at 37 °C. The test tubes were centrifuged for 8 min at 800 rpm. The optical density of the supernatant was measured at 545 nm. The percentage of haemolysis was calculated as follows:
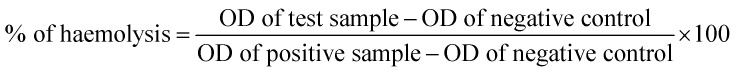


#### Thrombogenicity assay

The polymer films were hydrated by equilibrating them with saline water and they were kept at 37 °C in Petri dishes. ACD human blood (0.2 mL) was placed onto each film. Blood clotting was initiated by the addition of 0.02 mL of 0.1 M KCl solution followed by proper mixing with a Teflon stick. The clotting process was stopped by the addition of 5 mL of distilled water after 30 min. The clot formed was fixed in 5 mL of 3.6% formaldehyde solution for 5 min. The fixed clot was washed with distilled water, blotted between tissue paper and weighed.

### Cellular studies

#### Cell culture studies

The mouse mesenchymal stem cell line, C3H10t1/2 (NCCS Pune, India), was used for all experiments. The cells were cultured in 25 cm^2^ flasks at 37 °C in a humidified atmosphere with 5% CO_2_. Dulbecco's Modified Eagle's Medium (DMEM) high glucose medium together with 10% foetal bovine serum (FBS) and 1% antibiotic–antimycotic solution was used for culturing cells. The cells were seeded onto samples at an equal density of 2 × 10^3^ cells per surface (10 × 10 mm^2^) for all the cell-based assays.

#### Specimen for cell culture studies

The films of PVC and its various derivatives were prepared *via* a solution casting method in small Petri dishes. The prepared films were placed between two Teflon sheets and clamped for 10 min for obtaining the plane surface of materials. The cured specimens were removed from the moulds and their edges were smoothened with emery paper. The specimens were stored at room temperature. A specimen size of 4 × 4 mm^2^ was selected for in the *vitro* cell viability studies, while a specimen size of 10 × 10 mm^2^ was selected for the cell adhesion studies. Before performing the cell-based studies, the specimens were washed with isopropanol to remove the attached debris. For surface sterilization, each specimen was washed thrice with phosphate buffered saline (pH ∼ 7.2), kept in 70% alcohol and exposed to UV light for 8 h.

#### Cell adhesion

The ability of the samples to support cell adhesion was determined by staining the cells adhered to their surfaces with crystal violet. The cells were seeded on the surface of the samples at an equal density and incubated at 37 °C in a humidified atmosphere with 5% CO_2_ for 4 h. Prior to the addition of dye, the culture medium was aspirated, the cells were washed twice with cold phosphate buffered saline (PBS) pH 7.2 and fixed using a 4% formaldehyde solution. After the addition of the dye, the cells were incubated at room temperature for 30 min and then washed three times with cold PBS. Endogenous crystal violet was then extracted using absolute methanol and the absorbance of the solution was measured at 544 nm using a Fluostar optima (BMG Labtech, Germany) microplate reader. The cells adhered to the surface of the samples were quantified using the formula
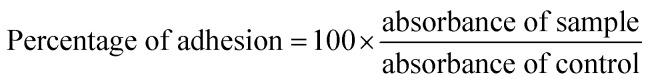


#### Cell viability

The MTT assay is a colorimetric test for measuring the activity of enzymes that reduce 3-[4,5-dimethylthiazol-2-yl]-2,5 diphenyltetrazolium bromide, (MTT) to formazan, giving a purple colour. The cytotoxicity of the samples was assessed using the MTT assay as described previously.^59^ 2 × 10^3^ cells in 200 μL of medium were seeded in each well and incubated for 24 h at 37 °C in a humidified CO_2_ incubator. Then supernatant medium including cell debris was replaced with fresh medium and the volume was maintained at 200 μL in each well. The samples were cut into small pieces (4 × 4 mm^2^) and released into each well after sterilization with 70% ethanol followed by UV exposure for 8 h and incubated for 1, 3 and 5 days in 96-well plates separately. After incubation, the supernatant medium of each well was discarded and 50 μL of MTT solution at 0.5 mg mL^−1^ in DMEM without FBS was added and incubated in the dark for 4 h at 37 °C. After 4 h, the MTT-containing medium was aspirated and 100 μL DMSO (Merck, India) was added to solubilize the formazan. The viable cells on the surface of the samples were quantified spectrophotometrically by measuring the absorbance at 570 nm using a microplate reader (iMark, Biorad). The percentage cell viability was calculated using the following formula:
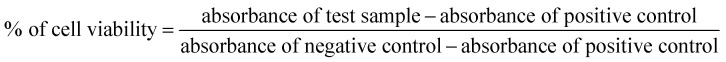
where, the negative control was the cells incubated with medium alone, positive control was cells incubated with medium and 10% DMSO and S is the test sample.

#### Fluorescence studies

The effect of pure polymer and its nanohybrids on cell proliferation was studied through fluorescence microscopy, where the cells were seeded in 24-well plates at the density of 1 × 10^4^ cells per mL and incubated for 24 h at 37 °C. The supernatant medium including cells debris was replaced with fresh medium in each well and the sterilized samples were released in triplicate and incubated for another 24 hours at 37 °C. Then, the samples were removed, washed thrice using 10 mM PBS, stained with fluorescent dye, acridine orange and ethidium bromide (Sigma, India) (100 μg mL^−1^ each) and incubated in the dark for 30 min at room temperature. Images were taken using a fluorescence microscope (Dewinter Technologies, India).

#### Statistical analysis

Statistical analysis was performed on the mean of the data obtained from three independent experiments using GRAPH PAD PRISM for Windows. The results are expressed as mean values (±SE). Analysis of variance followed by Tukey's multiple comparisons test was performed for the haemolysis assay, cell adhesion and cell viability for multiple comparison tests in two way of ANOVA. In all cases, the *p* value was obtained from the ANOVA table and the conventional 0.0332 level was considered to express the statistical significance.

## Result and discussion

### Determination of haemolysis activity of functionalized PVC/LDH nanocomposites

**Fig. 1:**
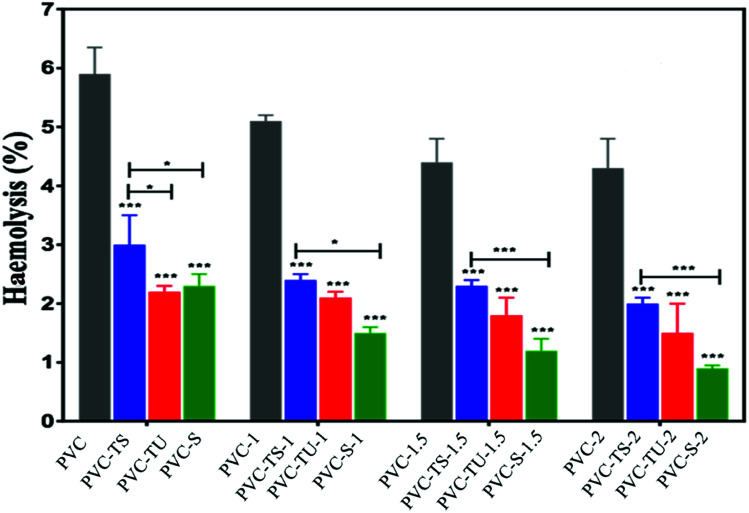
Haemolysis activities of the different forms of polymer-functionalized PVC composites. The difference was considered statically significant with *p < 0.033, **p < 0.0021, ***p < 0.0002, ****p < 0.0001 denoted with asterisks.

The release of haemoglobin is a primary quantifiable test to diagnose the biocompatibility of a material. The haemoglobin percentage represents the extent of RBC haemolysis when the material comes in contact with blood.^62^ Biocompatible materials must not encourage thrombosis, thromboembolism, antigenic reaction, and obliteration of blood constituents when they come into contact with blood.^63^ Therefore, blood biocompatibility is the most important test for biomaterials. Accordingly, we performed haemolysis tests for all the functionalized polymer nanocomposites.^64^Fig. 1 describes the result after the incubation of blood cells with the polymer nanocomposites. Haemolysis occurs when blood cells come in contact with water, and the positive control showed 100% lysis of blood cells, while the negative control showed 0% lyses of RBC. Each absorbance value represents the mean of the values observed for triplicate sets.

According to Autian (1975),^70^ the maximum value of haemolysis for a biocompatible material is 5%, which was fulfilled by all the prepared nanocomposites, *i.e.*, PVC-TS, PVC-TU and PVC-S.^68^ Additionally, all the polymer nanocomposites exhibited a haemolysis value within the normal limit (below 5%). Fig. 1 shows that the haemolysis value for PVC-2 was nearly 4.4% and that for the PVC-TS-1, PVC-TS-1.5 and PVC-TS-2 composites was 2.7%, 3.7% and 4.1%, respectively. Furthermore, all the PVC-TU composites were found to exhibit better haemocompatibility, with PVC-1, PVC-TU-1.5 and PVC-2 showing close to 1.2%, 0.9% and 0.7% haemolysis, respectively. Notably, all the ionomer composites of PVC-S exhibited excellent haemolytic properties compared to all the other modified materials. Thus, the results indicate that all the ionomer composites are advanced biomaterials, which exhibit excellent haemolytic properties and therefore can be used as alternatives to the neat form of PVC.

### Determination of clot formation on functionalized PVC/LDH nanocomposites

**Fig. 2:**
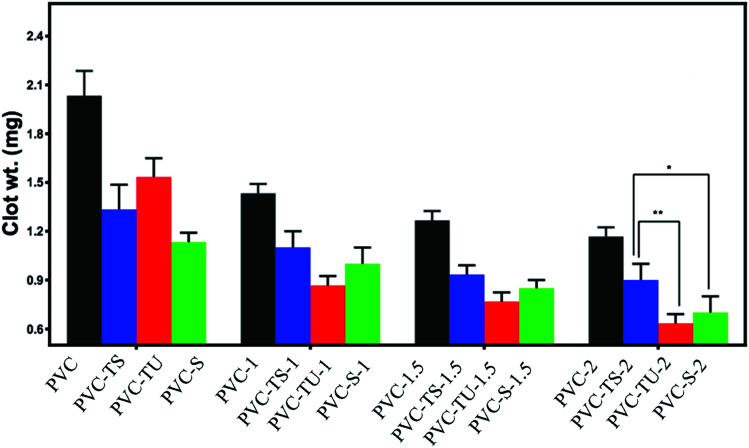
Thrombogenic properties of different functionalized PVC composites. The difference was considered statically significant with *p < 0.033, **p < 0.0021, ***p < 0.0002, ****p < 0.0001, as denoted with asterisks.

In addition to the haemolysis test, the thrombogenicity test is generally applied to measure the thrombogenic property of biomaterials. This is a straightforward, sensitive and standard measure of the compatibility of biomaterials with whole blood. Blood coagulation generally occurs when a foreign material comes into contact with blood. The initial blood response is the adsorption of blood protein followed by platelet adhesion and the activation of coagulation pathways, leading to thrombus formation.^65^ The thrombogenicity test was performed by placing ACD human blood on each functionalized polymer composite material. Coagulation started after the addition of KCl solution over the blood sample and it was terminated by mixing saline water after 30 min. The clot was fixed with a formaldehyde solution and weighed after completely drying. The assays were performed in triplicate and the data is summarized in Fig. 2. The functionalized PVC composites showed significantly less clot formation. With an increase in the LDH content in the polymeric matrix, clot formation decreased.^67^ Notably, the hydrophilicity of the material directly corresponds to their improved biocompatibility.

In addition, several studies suggest that biomaterials with a positively charged surface promote thrombogenesis when exposed to blood, while negatively charged biomaterials tend to suppress the thrombogenesis process,^5^ which is most likely because blood cells and platelets have a net negative charge on their surface.

### Cell adhesion and proliferation on functionalized PVC/LDH nanocomposites

**Fig. 3:**
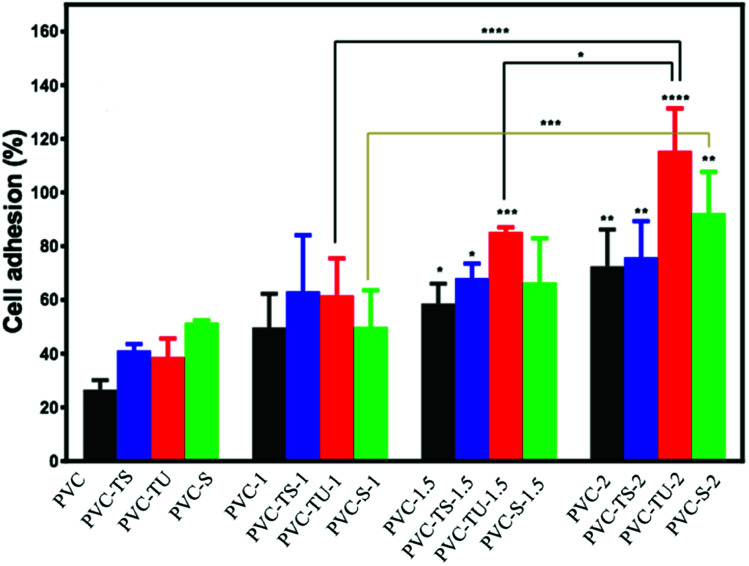
Cell adhesion of mouse mesenchymal stem cells with the different functionalized PVC/LDH nanocomposites. The difference was considered statically significant with *p < 0.033, **p < 0.0021, ***p < 0.0002, ****p < 0.0001, as denoted with asterisks.

Since in this study we evaluated the potential application of functionalized PVC/LDH nanocomposites in biomedical devices, it was essential to observe the cellular compatibility of the fabricated biomaterials, especially in terms of biocompatibility parameters such as adhesion and proliferation of cells on the nanocomposites films. Mouse mesenchymal stem cells are generally recommended as the reference cell type for cytotoxicity testing of biomaterials. All the functionalized PVC/LDH composites supported cellular adhesion under the standard conditions. Fig. 3 shows the percentage of stem cells adhered to the composites of PVC/LDH, PVC-TS/LDH, PVC-TU/LDH and PVC-S/LDH polymers after 4 h treatment. A polystyrene tissue cultured Petri dish (without sample) was used as a control in all cases. All the functionalized polymer ionomer composites showed an appreciably superior level of adhesion percentage. The level of cellular adhesion was found to increase in the polymer composites with an increase in the weight percentage of LDH. Cell adhesion was particularly more favorable with PVC-TU-1.5 and PVC-TU-2 because thiourea possesses a significant amount of amino moieties. Besides, PVC-2, PVC-TS-2 and PVC-S-2 showed a relatively similar range of cellular adhesion on their surfaces. Non-receptor-mediated cell adhesion on artificial materials indicates non-specific cell-material interactions *via* weak chemical bonding, such as hydrogen bonding, electrostatic, polar and ionic interactions, between various molecules on cell the membrane and functional chemical groups on the polymers.^71^

**Fig. 4:**
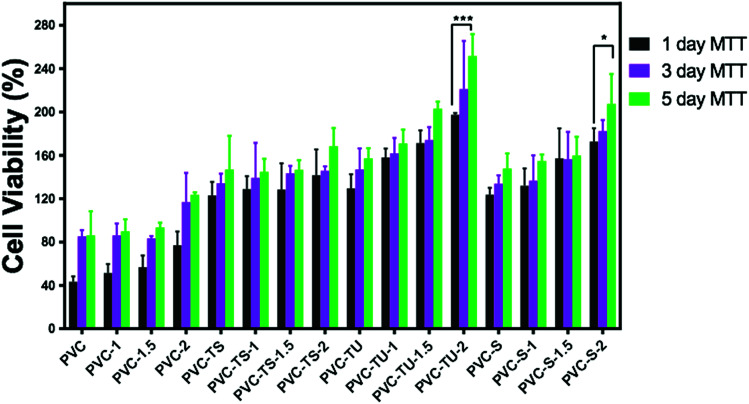
MTT assay of mouse mesenchymal stem cells with the different functionalized PVC/LDH nanocomposites. The difference was considered statically significant with *p < 0.033, **p < 0.0021, ***p < 0.0002, ****p < 0.0001, as denoted with asterisks.

To determine effects of the functional polymer nanocomposites on metabolic activity, the MTT test was performed. The cytotoxicity of the polymeric materials after their incubation with cells for 1, 3 and 5 days was observed in a culture medium. The cytotoxicity was measured by determining the cellular viability using the MTT assay. Fig. 4 presents a plot of the viability percentage of mouse mesenchymal stem cells, which shows significantly lower levels of cytotoxicity in the case of the functionalized polymer composites materials. The viability of the cells seeded on a bare tissue culture grade polystyrene Petri dish was considered as a control. It was observed that despite the decrease in the percentage of viable cells with an increase in the extract concentration, none of the tested materials appeared completely cytotoxic for mouse mesenchymal stem cells. Fig. 4 describes the cell viability of PVC and its various composites. After 1 day, the cell viability was found to be ∼43% for PVC, which increased significantly to ∼51%, ∼56%, and 76% for PVC-1, PVC-1.5, and PVC-2, respectively. However, after 3 days of culture, some changes were observed in the results. The viability with the PVC-1 and PVC-1.5 polymer composites did not increase as much as that of PVC-2, which was observed to be around 85% in PVC and 86%, 83%, and 116% for PVC-1, PVC-1.5, and PVC-2, respectively. The same pattern was observed after 5 days, where culture maintained its growth and the viability increased to 86% in PVC and 89%, 93% and 123% in PVC-1, PVC-1.5 and PVC-2, respectively.

PVC-TS showed better cell viability than PVC.^68^Fig. 4 reveals that PVC-TS showed 123% cell viability, which increased to 128%, 128% and 141% in PVC-TS-1, PVC-TS-1.5, and PVC-TS-2, respectively. The cell viability increased continuously even after the third day to 134% in PVC-TS, 139% in PVC-TS-1, 143% in PVC-TS-1.5 and 145% in PVC-TS-2. On the fifth day, it was 146% in PVC-TS, 144% in PVC-TS-1, 146% in PVC-TS-1.5 and 168% in PVC-TS-2.

The behavior of mouse mesenchymal stem cells on PVC-TU materials is always different because it contains zwitterionic groups, which favour the growth of these cells significantly. From the results, the different surfaces maintained the culture, as depicted Fig. 4. It can be observed that the initial cell viability with PVC-TU was 129%, which significantly increased to 158% in PVC-TU-1, 171% in PVC-TU-1.5 and 197% in PVC-TU-2 polymer nanocomposites. That observed on the third day was also better with 147%, 161%, 174% and 221% in PVC-TU, PVC-TU-1, PVC-TU-1.5 and PVC-TU-2, respectively. Similarly to the above trend, after the fifth day the cell growth remarkably increased to 157%, 170%, 202% and 251% in PVC-TU, PVC-TU-1, PVC-TU-1.5 and PVC-TU-2, respectively.

The cell viability was found to be notably greater than before, with 123%, 132% 157%, and 172% in PVC-S, PVC-S-1, PVC-S-1.5 and PVC-S-2, respectively, compared to PVC after one day of incubation.^58^ It increased to 133%, 136%, 156%, and 182% in PVC-S, PVC-S-1, PVC-S-1.5 and PVC-S-2 on the third day then to 147%, 154%, 159%, and 207% on the fifth day, respectively. Fischer *et al.* (2003) investigated the cell viability of different polycations and the magnitudes of the cytotoxic effects of all the polymers were found to be time and concentration dependent.^5^

### Fluorescence analysis of apoptotic cells

**Fig. 5:**
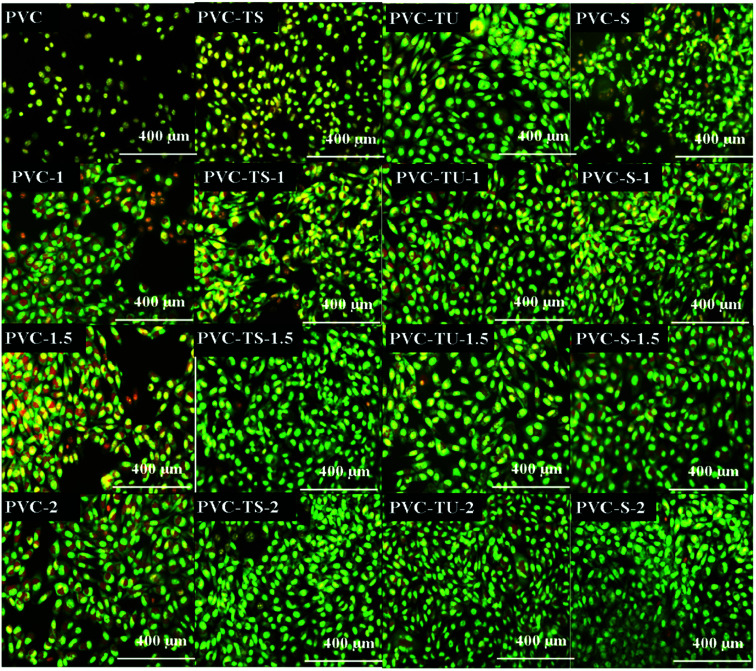
Morphological observation of the mMSCs grown on the different functionalized polymer nanocomposite surfaces for 24 h. Cells were cultured in direct contact with the various samples and analysed with a fluorescence microscope.

The biocompatibility study remains incomplete without the observation of the cell nuclei because it is important to determine the reason for cell death with biomaterials, *i.e.*, whether it occurs through apoptosis or necrosis. We observed a significant number of apoptotic nuclei in the mouse mesenchymal cells after 24 h of incubation with the functionalized polymer composites and their constituents. Further, it is important to assess whether cells can recover after damage or progress to necrosis due to the resulting inflammatory reaction and cellular injury. Fig. 5 shows the different characteristics of the cells after exposure to the dye, where the healthy and viable cells exhibit normal nuclei with a very good reticular pattern of green stain in the nuclei and red/orange granules in the cytoplasm.

Acridine orange is a vital dye that stains both live and dead cells. Ethidium bromide stains only cells that have lost membrane integrity. Live cells appear uniformly green. Early apoptotic cells stain green and contain bright green dots in their nuclei as a consequence of chromatin condensation and nuclear fragmentation. Late apoptotic cells also incorporate ethidium bromide and therefore stain orange, but, in contrast to necrotic cells, late apoptotic cells show condensed and often fragmented nuclei. Necrotic cells stain orange, but have a nuclear morphology resembling that of viable cells, with no condensed chromatin.^5,71^

Therefore, according to Fig. 5, PVC had poor adherence with no growth of mouse mesenchymal stem cells, but with the composites of PVC, cell growth increased. PVC-1 favoured cell growth with some dead cells and PVC-1.5 show more cells on its surface with a few apoptotic cells. In contrast, PVC-2 had more growth and much less apoptotic cells on its surface. PVC-TS exhibited fairly poor cell growth but was better than PVC. Also, an increase in the LDH concentration also increased the cell growth, where PVC-TS-1 had favorable growth with a few apoptotic cell, while PVC-TS-1.5 and PVC-TS-2 showed more cell growth and less apoptotic cells. PVC-TU showed favorable condition for cells and its composites provided a healthy environment. As the LDH concentration increased, cell growth increased, as exhibited by PVC-TU-1, 1.5 and 2. PVC-S itself showed cytotoxicity, including dotted nuclei in the apoptotic stage. However, in the PVC-S composites, as the LDH concentration increased, cell growth was promoted and less dead cell appears on their surface.

## Conclusions

This work demonstrated the influence of nanoclay with different functional groups on the characteristics of the PVC surface and the resulting biocompatibility properties. For this purpose, functionalized forms of PVC were fabricated using thiosulphate, thiourea and sulphate *via* a nucleophilic substitution reaction using a phase transfer catalyst together with the synthesis of LDH by *via* a co-precipitation method. The results revealed that the functionalized polymers are hydrophilic in nature, show reduced haemolytic activity and support cellular adhesion significantly. The addition of LDH in these functionalized PVC rendered them more hydrophilic, which improved their biocompatibility remarkably. Further research including *in vivo* testing for improving the biocompatibility of the surface-modified PVC polymers and their composites is necessary to fully authenticate their possible uses in biomedical-related applications.

## Conflicts of interest

There are no conflicts to declare.

## Supplementary Material
